# Determinants of infant breastfeeding practices in Nepal: a national study

**DOI:** 10.1186/s13006-019-0208-y

**Published:** 2019-04-03

**Authors:** Shiva Bhandari, Andrew L. Thorne-Lyman, Binod Shrestha, Sumanta Neupane, Bareng Aletta Sanny Nonyane, Swetha Manohar, Rolf D. W. Klemm, Keith P. West

**Affiliations:** 1 PoSHAN Study Team, Lalitpur, Nepal; 2 Department of Health Promotion, Education and Behavior, Arnold School of Public Health, University of South Carolina, Columbia, SC USA; 3 Center for Human Nutrition, Department of International Health, Johns Hopkins Bloomberg School of Public Health, Baltimore, MD USA; 4 Helen Keller International, New York, NY USA

**Keywords:** Breastfeeding, Colostrum, Infant, Prelacteal feeding, Nepal

## Abstract

**Background:**

Optimal breastfeeding practices, reflected by early initiation and feeding of colostrum, avoidance of prelacteal feeds, and continued exclusivity or predominance of breastfeeding, are critical for assuring proper infant nutrition, growth and development.

**Methods:**

We used data from a nationally representative survey in 21 district sites across the Mountains, Hills and Terai (southern plains) of Nepal in 2013. Determinants of early initiation of breastfeeding, feeding of colostrum, prelacteal feeding and predominant breastfeeding were explored in 1015 infants < 12 months of age. Prelacteal feeds were defined as food/drink other than breast milk given to newborns in first 3 days. Predominant breastfeeding was defined as a child < 6 months of age is mainly breastfed, not fed solid/semi-solid foods, infant formula or non-human milk, in the past 7 days. Adjusted prevalence ratios (APR) and 95% confidence intervals (CI) were estimated, using log Poisson regression models with robust variance for clustering.

**Results:**

The prevalence of breastfeeding within an hour of birth, colostrum feeding, prelacteal feeding and predominant breastfeeding was 41.8, 83.5, 32.7 and 57.2% respectively. Compared to infants not fed prelacteal feeds, infants given prelacteal feeds were 51% less likely to be breastfed within the first hour of birth (APR 0.49; 95% CI 0.36, 0.66) and 55% less likely to be predominantly breastfed (APR 0.45; 95% CI 0.32, 0.62). Infants reported to have received colostrum were more likely to have begun breastfeeding within an hour of birth (APR 1.26; 95% CI 1.04, 1.54) compared to those who did not receive colostrum. Infants born to mothers ≥ 20 years of age were less likely than adolescent mothers to initiate breastfeeding within 1 hour of birth. Infants in the Terai were 10% less likely to have received colostrum (APR 0.90; 95% CI 0.83, 0.97) and 2.72 times more likely to have received prelacteal feeds (APR 2.72; 95% CI 1.67, 4.45) than those in the Mountains.

**Conclusions:**

Most infants in Nepal receive colostrum but less than half initiate breastfeeding within an hour of birth and one-third are fed prelacteal feeds, which may negatively affect breastfeeding and health throughout early infancy.

**Electronic supplementary material:**

The online version of this article (10.1186/s13006-019-0208-y) contains supplementary material, which is available to authorized users.

## Background

Appropriate and optimal infant feeding is fundamentally important to assure adequate nutrition and growth during infancy. Optimal breastfeeding involves complementary feeding and overlapping practices of exclusive breastfeeding (breastmilk with no other foods or liquids) for the first 6 months of life, early inititiation of breastfeeding as soon as a child is born, feeding colostrum and avoiding prelacteal foods [1]. In Nepal and elsewhere throughout South Asia, suboptimal infant feeding practices have been associated with undernutrition, reflected by stunting and wasting, and mortality [2–4]. Practices such as early initiation of breastfeeding, avoiding prelacteal feeds, assuring intake of colostrum and maintaining exclusivity of breastfeeding in early infancy, represent critical exposures that benefit child growth and development [5, 6]. Exclusive breastfeeding up to 6 months of age and continuance of breastfeeding during the first [7] and second [8] years of life have been associated with increased linear growth and better cognitive development scores [9].

The World Health Organization (WHO) recommends that mothers practice exclusive breastfeeding for the first 6 months of life, followed by a timely introduction of appropriate complementary foods [10]. Early initiation of breastfeeding (i.e. within 1 h of birth) is recommended as the first critical step to ensure children receive colostrum, the “first milk” which is rich in nutrients and antibodies essential for rapid adaptation to postnatal life. Early suckling can also facilitate success with subsequent breastfeeding practices by stimulating the release of prolactin, enabling the mother to produce more milk [11]. Yet, only two-thirds of mothers in Nepal are reported to exclusively breastfeed their infants in the past 24 h (66.1%) [12]. Concerns exist that, in Nepal, the prevalence of exclusive breastfeeding in early infancy may be in decline, as indicated by a slight reduction from about 70 to 66% between consecutive Demographic Health Surveys (DHS) from 2011 to 2016 [13].

In Nepal [14], elsewhere in South Asia [15–19] and in other regions [20, 21], colostrum may often be discarded, despite nutritional and immunological benefits it confers to newborns [22], and replaced by prelacteal feeds. Prelacteal feeding not only displaces breastmilk, but also can disrupt the priming of the gastrointestinal tract [23] and may introduce pathogens that increase the risk of illness [24]. Consequent delay in establishing breastfeeding has been shown to predispose infants to a higher risk of mortality in a dose response fashion [3].

In South Asia, including Nepal, despite the increased policy and programmatic investment in behavior change communication to promote optimal feeding practices for infants [25], achieving the targets set by WHO is proving to be challenging [26]. Small area studies have been conducted in Nepal to identify factors related to infant feeding practices, mothers’ knowledge on how long a child should be given only breast milk, perceptions about the benefits of breastfeeding, socioeconomic status, and mothers’ education [27–29] that may help guide more effective breastfeeding promotion. However, there remains uncertainty about the generalizability of these findings nationally. The present paper presents prevalence estimates for four breastfeeding practices as assessed in a nationally representative sample of infants (< 12 months of age) in Nepal and examines factors that are associated with these feeding practices at individual, household and community levels.

## Methods

### Study design

**Fig. 1:**
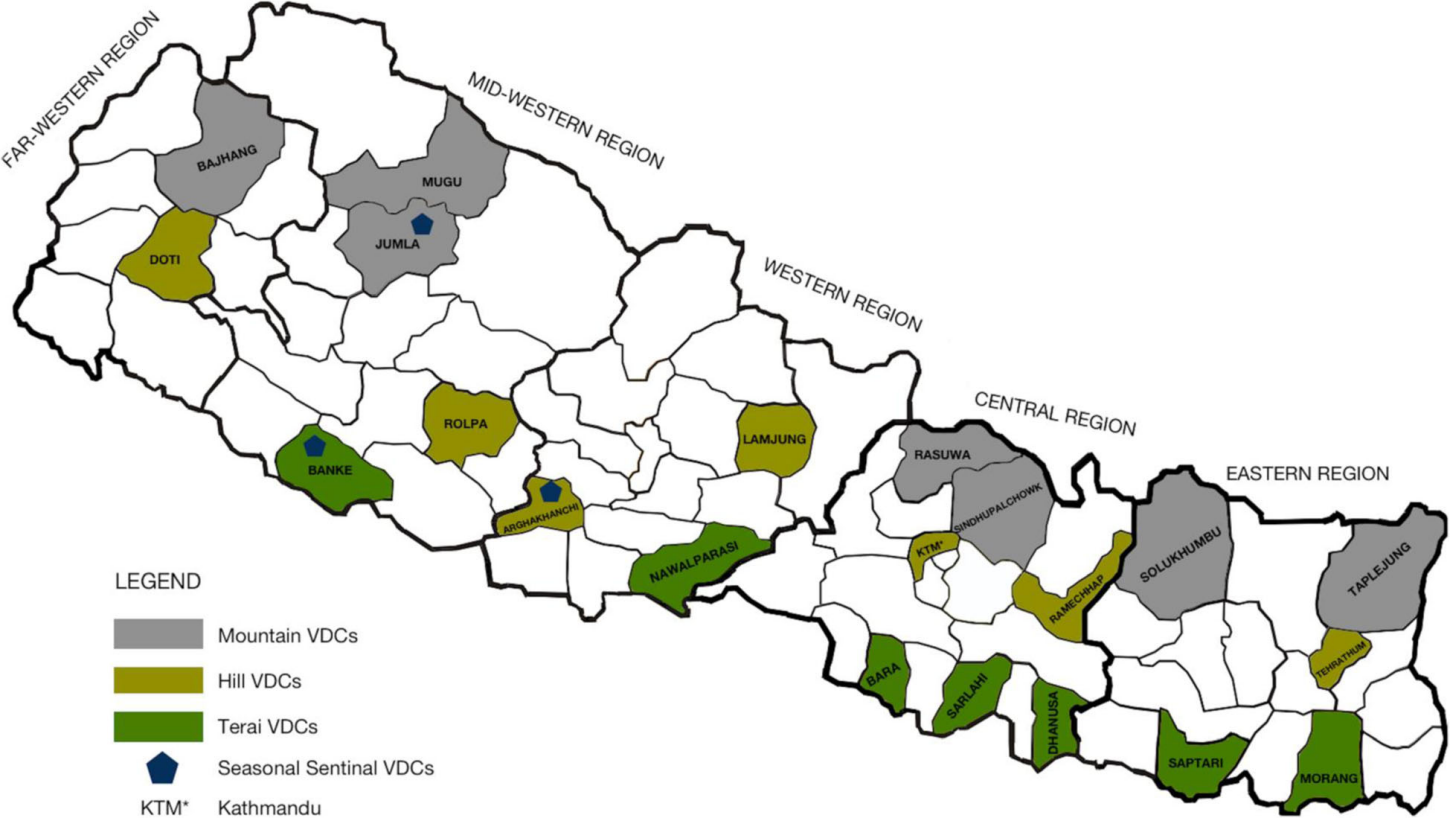
PoSHAN Community Study districts, Nepal, 2013 (adapted with permission from [30])

Data used for this analysis was collected during a national survey (the PoSHAN Community Study) conducted from May to July 2013. The design of the study is described in detail elsewhere [30]. In brief, systematic random sampling following a random start was carried out to select village development committees (VDCs) from a West to East listing of all contiguous VDCs in each agro-ecological zone. Seven VDCs, each from different districts, across each zone (a total of 21 VDCs in 21 districts) were selected. Wards were listed by population size in each VDC (*n* = 9) from which three were systematically selected following a random start. In total, 63 wards were sampled (21 × 3), in which all households were visited. The study districts are shown in Fig. 1. The households were eligible for the study if there were children less than 5 years of age or women without children who were married within the past 2 years. Heads of household and mothers were consented and invited to participate in the survey. Information was collected on household characteristics, mothers and children under 5 years of age. However, for the present analysis of breastfeeding practices and risk factors, we include data only from households with infants under 12 months of age at the time of the survey to minimize recall bias with respect to early infant feeding practices that may exist among mothers of older children [31].

### Data collection

In each sampled VDC, 21 field teams, each consisting of three experienced interviewers and one supervisor were hired from a local research firm (New ERA Pvt. Ltd). Field teams were trained and standardized in obtaining informed consent and conducting interviews over a period of a month. Questionnaires were pre-tested across agro-ecological zones and interviews were conducted primarily in Nepali. The final questionnaires were in Nepali and translation was done where required. In certain VDCs, as appropriate, interviews were conducted in Awadhi, Maithilee and Bhojpuri languages. Data collection was monitored in the field by a trained supervisor and quality control team. Household information was obtained by interviewing the head, whereas maternal and child levels of information were obtained by interviewing mothers. Data were collected using paper forms that were checked in the field for legibility and completeness and transmitted to a data entry center in Kathmandu for checking, entry and range and other consistency checks were undertaken.

### Outcome variables

Among infants, field staff inquired about early initiation of breastfeeding (within 1 h of birth), feeding of colostrum and any prelacteal feeds, and predominant breastfeeding as study outcomes. We used predominant breastfeeding instead of exclusive breastfeeding as data was not collected on intake of water-based fluids by infants in the past 7 days [32]. Use of predominant breastfeeding as an indicator is helpful to understand breastfeeding practices in the absence of an exclusive breastfeeding indicator. However, the rates from these two exclusive breastfeeding and predominant breastfeeding indicators cannot be directly comparable and one should clearly explain which rate is being reported. The definitions and measurement approaches for the outcome variables were based on the WHO Infant and Young Child Feeding (IYCF) indicator guide, with the exception of feeding colostrum and prelacteal feeds, which we appended to adopted WHO indicator variables [32]. Definitions of these outcomes are provided below:

#### Breastfeeding within one hour of birth

Mothers of infants were asked how soon after birth the child was put to the breast and enumerators coded responses into four categories (< 1 h, 1 h to < 24 h, two or more days, never breastfed). We dichotomized these responses using a cutoff of < 1 h, for consistency with the WHO indicator.

#### Feeding Colostrum

Colostrum was defined as the first yellowish human breast milk produced after giving birth. Mothers were asked whether the child was fed colostrum.

#### Prelacteal feeds

Prelacteal feeds were defined as foods or drinks other than human breast milk given to newborns in the first 3 days of life. Mothers giving any prelacteal feeds in the first 3 days of life were categorized as prelacteal feeders.

#### Predominant breastfeeding

WHO defines predominant breastfeeding as a condition where a child < 6 months of age is mainly breastfed, not fed solid/semi-solid foods, infant formula or non-human milk, and may or may not have received water-based fluids (water, water-based drinks, juice, ORS, ritual fluids, vitamins/minerals/medicines) in the past 24 h. However, in this study the recall period was the past 7 days. Also, as this practice is age-dependent, analysis was stratified by age, and restricted to infants < 6 months of age. Because we did not capture feeding information for the entire first 6 months of life, there is no overlap with prelacteal feeding.

### Covariates

To explore determinants of the breastfeeding practices, we categorized explanatory variables into child, maternal, household, and community-level factors. The selection of these variables was informed by a review of the literature and factors that have been shown or hypothesized to be associated with breastfeeding practices [33, 34].

Child-level factors included gender, age and birth order (first born child vs second or later born child) which were treated as categorical variables.

Maternal-level factors included age, educational attainment, occupation, antenatal care (ANC) and postnatal care visit, knowledge about recommended breastfeeding practices and women’s empowerment. We developed a women’s empowerment variable based on a simple 14 item scale of participation in critical household decisions related to expenditures, production, parenting, and autonomy. This was dichotomized at the upper 25th percentile. Mothers who were in the upper 25 percentile with a score of > = 9 were considered more empowered. All the variables were treated as categorical.

Household-level factors included presence of father in the household; father’s education and occupation, household head’s gender, education and occupation; ethnicity/caste; household cultivable land size and wealth quintiles. Principal component analysis was conducted based on a list of durable asset and land ownership using the method described by Ruestein and colleagues [35]. Quintiles of this scale were then created.

Community-level factors included agro-ecological zone (Mountain, Hills and Terai) and VDC infrastructure. A VDC was considered more developed if it had one of the following: presence of paved roads, PHC/hospitals, permanent bazar or secondary/higher secondary school. All the variables were treated as categorical.

### Statistical analysis

We used prevalence ratios (PR), a measure that is analogous to the risk ratios of cohort studies, to evaluate the associations between determinants and breastfeeding practices [36, 37]. Prevalence ratios and 95% confidence intervals (95% CI) were derived using Poisson regression with robust variance to account for clustering by wards [38, 39]. In all multivariable adjusted models, we included mother’s education and visit by female community health volunteers (FCHVs) for antenatal care as we identified these variables a priori as important potential covariates because mother’s education is an important predictor of breastfeeding practices [40] and FCHVs are heavily involved in community based infant and young child feeding programs [41]. We then used a two-step-approach to make decisions about the selection of additional variables. Unadjusted relationships were first examined (Model 1), and those variables with a *p* value < 0.2 were retained in the first set of multivariable models (Model 2), that were run separately for each grouping of covariates; i.e., by levels of child, maternal, household and agro-ecological zone. The final multivariable model (Model 3) included only those variables that had a *p* value < 0.2 in Model 2. The threshold used to determine statistical significance for interpretation of all models was a *p* - value < 0.05. Only variables retained in each model are presented in tables or the results. All statistical analyses were performed using Stata version 13.1 (StataCorp, Texas).

### Ethical approval

Participants were briefed about the purpose and assessment activities of the study. Participation was voluntary and agreement to participate was documented as an oral consent. As some of the respondents were illiterate, we could not use informed written consent. Ethical approval for the study was provided by the Nepal Health Research Council, an autonomous body, under the Ministry of Health, Government of Nepal, and the Institutional Review Board at the Johns Hopkins Bloomberg School of Public Health, Baltimore, MD.

## Results

### General characteristics of the participants and breastfeeding practices

Mothers of 1015 infants were interviewed. Slightly more than half of the infants were male (53.5%) and aged 6 to 11.9 months (54.9%). Most interviewed mothers were 20 to 29 years of age (69.0%), while 14.7% were adolescents (15 to 19 years of age). Nearly half of the mothers (47.4%) had never attended school, 13.3% had some primary education and 39.8% had at least secondary education. Three quarters of mothers (76.4%) were not formally employed and 13.5% were employed in agriculture. More than half of the infants resided in the Terai (58.8%), and a quarter in Hills (25.4%) with the remainder (15.8%) in the Mountains (Table 1).

**Table 1:** General characteristics of study population and explanatory factors

	*n* (%)
Child level factors (*n* = 1015)	
Child’s gender	
Male	543 (53.5)
Female	472 (46.5)
Child’s age (in months), mean (SD)	5.9 (3.4)
Child’s age (in months)	
0–5.9	458 (45.1)
6–11.9	557 (54.9)
Birth order	
First born child	540 (53.2)
Second or later born child	475 (46.8)
Maternal level factors (*n* = 1015)	
Mother’s age (in years), mean (SD)	24.4 (5.4)
Mother’s age (in years)	
15–19.9	149 (14.7)
20–29.9	700 (69.0)
> = 30	166 (16.4)
Mother’s education	
None	481 (47.4)
Some primary	135 (13.3)
Secondary and above	398 (39.3)
Mother’s occupation	
Unemployed	775 (76.4)
Agriculture	137 (13.5)
Other	103 (10.2)
Visit by FCHV for ANC	
No	913 (90.0)
Yes	102 (10.1)
Visit by more highly trained healthcare providers ^a^ for ANC	
No	976 (96.2)
Yes	39 (3.8)
Visit to health facilities for ANC	
No	390 (38.4)
Yes	625 (61.6)
Visit by FCHV for postnatal care	
No	907 (89.4)
Yes	108 (10.6)
Visit by more highly trained healthcare providers ^a^ for PNC	
No	963 (94.9)
Yes	52 (5.1)
Visit to health facilities for postnatal care	
No	663 (65.3)
Yes	352 (34.7)
Maternal knowledge present on:	
Exclusive breastfeeding for infants up to 6 months of age	
No	368 (36.3)
Yes	647 (63.7)
Breastfeeding for children during diarrhea	
No	809 (79.7)
Yes	206 (20.3)
Mothers’ empowerment (mean, SD)	5.5 (3.1)
Mothers’ empowerment (scale: 0–14, Median = 5)	
< = 8 (less empowered)	830 (81.8)
> = 9 (more empowered)	185 (18.2)
Household level factors (*n* = 1015)	
Presence of father at home	
No	325 (32.0)
Yes	690 (68.0)
Father’s education (among those present at home)	
None	154 (22.3)
Some primary	149 (21.6)
Secondary and above	387 (56.1)
Household head’s gender	
Male	808 (79.6)
Female	207 (20.4)
Household head’s education	
None	485 (47.8)
Some primary	191 (18.8)
Secondary and above	339 (33.4)
Household head’s occupation	
Unemployed ^a^	122 (12.0)
Wage employment	190 (18.7)
Business/self-employment	212 (20.9)
Salaried worker	101 (10.0)
Agriculture	389 (38.4)
Ethnicity/Caste	
Upper caste (Brahmins, chhetris)	218 (21.5)
Disadvantaged non-dalit Terai caste	341 (33.6)
Janajatis	228 (22.5)
Lower caste (Dalits, religious minorities)	228 (22.5)
Household wealth quintile	
1 (Poorest)	203 (20.0)
2	199 (19.6)
3	204 (20.1)
4	206 (20.3)
5 (Richest)	203 (20.0)
Father’s occupation (among those present at home)	
Unemployed ^a^	19 (2.8)
Wage employment	195 (28.3)
Business/self-employment	178 (25.8)
Salaried worker	124 (18.0)
Agriculture	174 (25.2)
Cultivable land size (in Ha)	
Landless (< 0.1)	424 (41.8)
Small size (> = 0.1 & < 0.5)	261 (25.7)
Large size (> = 0.5)	330 (32.5)
Contextual factors (*n* = 1015)	
Agro-ecological zones	
Mountain	160 (15.8)
Hill	258 (25.4)
Terai	597 (58.8)
Ward infrastructure is more developed	
No	502 (49.5)
Yes	513 (50.5)
Breastfeeding practices	
Prelacteal feeds given	
Not fed	677 (67.3)
Fed	329 (32.7)
Breastfed within one hour after birth	
No	588 (58.2)
Yes	423 (41.8)
Colostrum fed	
No	167 (16.5)
Yes	844 (83.5)
Predominant breastfeeding (children < 6 months) (*n* = 458)	
No	196 (42.8)
Yes	262 (57.2)

^a^“More highly trained healthcare providers” includes other govt health workers (MCHW/VHW, HA/AHW, Nurse/Midwife), doctors/pharmacists and NGO health workers; “Unemployed” includes student, non-earning occupation as well as non-working

Breast milk was introduced within 1 h of birth in 41.8% of infants. One-third of infants (32.7%) were reported to have received prelacteal feeds as their first food, 83.5% were fed colostrum, and predominant breastfeeding (PBF) was practiced by 57.2% of interviewed mothers infants less than 6 months of age (Table 1).

### Determinants of breastfeeding within one hour of birth (Table 2)

In multivariable adjusted models exploring predictors of breastfeeding within 1 h of birth, associations with maternal age were apparent: compared with infants of younger mothers (< 20 y) those born to mothers 20–29 and ≥ 30 years of age were 19% (adjusted prevalence ratio [APR] 0.81; 95% CI 0.68, 0.95) and 39% (APR 0.61; 95% CI 0.43, 0.87) less likely to have reported breastfeeding within an hour after birth. Mothers who had agriculture as an occupation were also 28% more likely to have breastfed their children within one hour of birth compared to mothers who were unemployed (APR 1.28; 95% CI 1.02, 1.60).

**Table 2:** Determinants of breastfeeding within one hour of birth among infants in Nepal, 2013^a,b,c^

Determinants	*n* (%)	Breastfed within an hour *n* (%)	Model 1 (Unadjusted PR) PR (95% CI)	Model 3^d^ (Adjusted PR) APR (95% CI)
Overall	1011 (100)	423 (41.8)		
Child factors				
Child’s gender				
Male	541 (53.5)	222(41.0)	1.00	–
Female	470 (46.5)	201(42.8)	1.03 (0.91–1.18)	
Child’s birth order				
First born	538 (53.2)	210 (39.0)	1.00	–
Second or later born	473 (46.8)	213 (45.0)	1.18 (1.00,1.38)*	
Child fed colostrum				
No	167 (16.5)	54 (32.3)	1.00	1.00
Yes	844 (83.5)	369 (43.7)	1.32 (1.07,1.64)*	1.26 (1.04,1.54)*
Child fed prelacteal feeds				
No	677 (67.3)	343 (50.9)	1.00	1.00
Yes	329 (32.7)	78 (23.7)	0.47 (0.35,0.63)**	0.49 (0.36,0.66)**
Predominant breastfeeding (Infant < 6 mo)				
No	196 (42.8)	75 (38.3)	1.00	–
Yes	262 (57.2)	130 (50.0)	1.33 (1.01,1.74)*	
Maternal factors				
Mother’s education				
None	479 (47.4)	200 (41.8)	1.00	1.00
Some primary	135 (13.4)	53 (39.3)	0.93 (0.72,1.2)	0.91 (0.70–1.19)
Secondary and above	396 (39.2)	170 (42.9)	1.00 (0.87,1.16)	1.01 (0.84–1.21)
Mother’s age (in years)				
15–19.9	149 (14.7)	68 (45.6)	1.00	1.00
20–29.9	697 (68.9)	299 (42.9)	0.93 (0.79,1.09)	0.81 (0.68–0.95)*
≥ 30	165 (16.3)	56 (33.9)	0.72 (0.53,0.97)*	0.61 (0.43–0.87)*
Mother’s occupation				
Unemployed	773 (76.5)	305 (39.5)	1.00	1.00
Agriculture	136 (13.5)	72 (52.9)	1.31 (1.04,1.64)*	1.28 (1.02–1.60)*
Other employment^e^	102 (10.1)	46 (45.1)	1.10 (0.85,1.43)	1.09 (0.83–1.42)
Visit by FCHVs for ANC				
No	909 (89.9)	382 (42)	1.00	
Yes	102 (10.1)	41 (40.2)	0.98 (0.78,1.24)	0.99 (0.77–1.27)
Visit by FCHVs for postnatal care				
No	903 (89.3)	371 (41.1)	1.00	1.00
Yes	108 (10.7)	52 (48.2)	1.17 (0.94,1.45)	1.12 (0.91–1.37)
Visit by more highly trained healthcare providers^e^ for postnatal care				
No	959 (94.9)	408 (42.5)	1.00	1.00
Yes	52 (5.1)	15 (28.9)	0.69 (0.49,0.99)*	0.72 (0.49–1.05)
Maternal knowledge on exclusive breastfeeding for infants up to 6 months of age				
No	368 (36.4)	136 (37)	1.00	1.00
Yes	643 (63.6)	287 (44.6)	1.17 (0.97,1.41)	1.19 (0.99–1.44)
Number of live births given				
1	380 (37.7)	149 (39.2)	1.00	1.00
> = 2	629 (62.3)	274 (43.6)	1.12 (0.96,1.30)	1.11 (0.85–1.43)
Household factors				
Ethnicity/Caste				
Upper caste	216 (21.4)	104 (48.2)	1.00	1.00
Disadvantaged non-dalit Terai caste	341 (33.7)	137 (40.2)	0.95 (0.76,1.20)	1.02 (0.81–1.27)
Janajatis	227 (22.5)	87 (38.3)	0.83 (0.67,1.04)	0.91 (0.73–1.14)
Lower caste^e^	227 (22.5)	95 (41.9)	0.93 (0.74,1.17)	0.95 (0.76–1.19)
Household wealth quintile				
1 (Poorest)	202 (20)	89 (44.1)	1.00	1.00
2	198 (19.6)	79 (39.9)	0.92 (0.73,1.14)	0.94 (0.75–1.18)
3	204 (20.2)	93 (45.6)	1.03 (0.8,1.33)	1.07 (0.82–1.38)
4	204 (20.2)	93 (45.6)	1.05 (0.82,1.34)	1.07 (0.79–1.45)
5 (Richest)	203 (20.1)	69 (34.0)	0.77 (0.57,1.06)	0.79 (0.55–1.12)
Occupation of household head				
Unemployed^e^	122 (12.1)	51 (41.8)	1.00	1.00
Wage employment	190 (18.8)	83 (43.7)	1.07 (0.83,1.39)	1.03 (0.81–1.33)
Business/self-employment	210 (20.8)	72 (34.3)	0.82 (0.58,1.17)	0.80 (0.57–1.13)
Salaried worker	101 (10)	57 (56.4)	1.32 (1.01,1.73)*	1.27 (0.94–1.71)
Agriculture	387 (38.3)	160 (41.3)	0.99 (0.73,1.34)	0.88 (0.65–1.19)
Contextual factors				
Agro-ecological zones				
Mountain	158 (15.6)	77(48.7)	–	1.00
Hill	256 (25.3)	111(43.4)		1.00 (0.79–1.26)
Terai	597 (59.1)	235(39.4)		1.03 (0.78–1.36)

^a^For interpretation purposes, a PR > 1 indicates children are more likely to be breastfed within an hour of birth and PR < 1 indicates children are less likely

^b^* *P*-value < 0.05, ** *P*-value < 0.001

^c^(Model 2 shown in Additional file 1)

^d^Model 3 included mother’s education and visit by FCHVs for ANC as a priori covariates plus all variables that were significant (*p* <  0.2) in the first set of multivariable models

^e^“Other employment” included wage employment, salaried worker and Business/self-employment. “More highly trained healthcare providers” includes government health workers (MCHW/VHW, HA/AHW, Nurse/Midwife), doctors/pharmacists and NGO health workers. “Lower caste” includes Dalits and religious minorities. “Unemployed” includes student, non-earning occupation as well as non-working

### Determinants of colostrum feeding (Table 3)

Several factors were associated with a slight but significant increased likelihood of feeding the newborn infant colostrum in multivariable adjusted models, including maternal age 20–29 y (vs. age < 20 y), greater women’s empowerment, a reproductive history that included an abortion in their lifetime, a large land holding and household wealth classified to be in the upper 40th percent of the nationally compiled index (vs. in the lowest fifth). Infants born to mothers in households where the heads were salaried workers or involved in agriculture were 9% less likely to be given colostrum (APR 0.91; 95% CI 0.84, 0.98) compared to the newborns into households whose head was unemployed. Newborns in the Terai were 10% less likely to receive colostrum than those born in the mountains (APR 0.90; 95% CI 0.83, 0.97).

**Table 3:** Determinants of feeding colostrum among infants in Nepal, 2013^a,b,c^

Determinants	*n*	Fed colostrum, *n* (%)	Model 1 (Unadjusted PR) PR (95% CI)	Model 3^d^ (Adjusted PR) APR (95% CI)
Overall	1011	844 (83.5)		
Child factors				
Child’s gender				
Male	541	448(82.8)	1.00	–
Female	470	396(84.3)	1.01 (0.96–1.06)	
Child’s birth order				
First born	538	455 (84.6)	1.00	–
Second or later born	473	389 (82.2)	0.99 (0.93–1.06)	
Breastfed within one hour of birth				
No	588	475 (80.8)	1.00	1.00
Yes	423	369 (87.2)	1.07 (1.01,1.13)*	1.06(1.01,1.11)*
Child fed prelacteal feeds				
No	674	584 (86.7)	1.00	1.00
Yes	329	255 (77.5)	0.92 (0.84,1.00)*	0.92(0.86,0.99)*
Predominant breastfeeding (Infant < 6 mo)				
No	196	166 (84.7)	1.00	–
Yes	260	217 (83.5)	1.00 (0.94,1.05)	
Maternal factors				
Mother’s education				
None	479	373 (77.9)	1.00	1.00
Some primary	135	115 (85.2)	1.08 (1–1.17)	1.05 (0.96–1.14)
Secondary and above	396	355 (89.7)	1.12 (1.06–1.19)**	1.04 (0.97–1.12)
Mother’s age (in years)				
15–19.9	149	113 (75.8)	1.00	1.00
20–29.9	697	595 (85.4)	1.12 (1.02–1.23)*	1.09 (1.00–1.19)*
≥ 30	165	136 (82.4)	1.05 (0.93–1.19)	1.07 (0.94–1.22)
Visit by FCHVs for ANC				
No	909	760 (83.6)	1.00	1.00
Yes	102	84 (82.4)	1.01 (0.91–1.13)	1 (0.91–1.11)
Visit by more highly trained healthcare providers^e^ for ANC				
No	972	816 (84.0)	1.00	1.00
Yes	39	28 (71.8)	0.91 (0.8–1.02)	0.94 (0.86–1.03)
Visit to health facilities for ANC				
No	388	308 (79.4)	1.00	1.00
Yes	623	536 (86.0)	1.08 (1.00–1.17)	1.07 (0.98–1.16)
Maternal knowledge on exclusive breastfeeding for infants up to 6 months of age				
No	368	285 (77.5)	1.00	1.00
Yes	643	559 (86.9)	1.08 (1.02–1.15)*	1.04 (0.98–1.09)
Women’s empowerment (scale: 0–14, Md = 5)				
≤ 8 (less empowered)	826	677 (82.0)	1.00	1.00
≥ 9 (more empowered)	185	167 (90.3)	1.07 (1.00–1.14)*	1.08 (1.01–1.15)*
Had abortions in lifetime				
No	972	806 (82.9)	1.00	1.00
Yes	39	38 (97.4)	1.12 (1.06–1.19)**	1.10 (1.02–1.17)*
Had miscarriage/stillbirths in lifetime				
No	847	713 (84.2)	1.00	1.00
Yes	164	131 (79.9)	0.94 (0.88–1.00)	0.93 (0.87–1.00)
Household factors				
Household head’s education				
None	484	381 (78.7)	1.00	1.00
Some primary	189	159 (84.1)	1.06 (0.98–1.14)	1.01 (0.94–1.09)
Secondary and above	338	304 (89.9)	1.12 (1.07–1.17)**	1.05 (1.00–1.10)
Household wealth quintile				
1 (Poorest)	202	158 (78.2)	1.00	1.00
2	198	156 (78.8)	1.02 (0.92–1.13)	1 (0.90–1.1)
3	204	162 (79.4)	1.02 (0.94–1.10)	0.97 (0.90–1.06)
4	204	183 (89.7)	1.17 (1.08–1.26)**	1.09 (1.01–1.19)*
5 (Richest)	203	185 (91.1)	1.17 (1.10–1.25)**	1.08 (1.00–1.18)
Occupation of household head				
Unemployed^e^	122	107 (87.7)	1.00	1.00
Wage employment	190	152 (80)	0.94 (0.86–1.03)	0.96 (0.89–1.05)
Business/self-employment	210	176 (83.8)	0.97 (0.90–1.05)	0.94 (0.88–1.00)
Salaried worker	101	88 (87.1)	0.98 (0.91–1.06)	0.91 (0.84–0.98)*
Agriculture	387	320 (82.7)	0.94 (0.88–1.02)	0.91 (0.84–0.98)*
Cultivable land size (in Ha)				
Landless (< 0.1)	421	343 (81.5)	1.00	1.00
Small size (≥ 0.1 & < 0.5)	260	216 (83.1)	1 (0.93–1.08)	1.06 (0.99–1.15)
Large size (≥ 0.5)	330	285 (86.4)	1.07 (1.01–1.14)*	1.12 (1.05–1.19)**
Contextual factors				
Agro-ecological zones				
Mountain	158	144 (91.1)	–	1.00
Hill	256	231 (90.2)		0.99 (0.93–1.05)
Terai	597	469 (78.6)		0.9 (0.83–0.97)*
Ward infrastructure is more developed				
No	502	394 (78.5)	1.00	1.00
Yes	509	450 (88.4)	1.09 (1.00–1.18)*	1.04 (0.96–1.13)

^a^Prevalence ratio: a PR > 1 indicates feeding of colostrum is more likely and PR < 1 indicates that feeding of colostrum is less likely

^b^*P-value < 0.05, **P-value < 0.001

^c^(Model 2 shown in Additional file 2)

^d^Model 3 included mother’s education and visit by FCHVs for ANC as a priori covariates plus all variables that were significant (*p* < 0.2) in the first set of multivariable models

^e^“more highly trained healthcare providers” includes government health workers (MCHW/VHW, HA/AHW, Nurse/Midwife), doctors/pharmacists and NGO health workers. “Unemployed” includes student, non-earning occupation as well as non-working

### Determinants of prelacteal feeding (Table 4)

Second or later born infants were 31% less likely than first born infants to have received prelacteal feeds (APR 0.69; 95% CI 0.55, 0.87). Infants born in the Terai were 2.7 times more likely to have been fed prelacteal feeds than those in the mountains (APR 2.72; 95% CI 1.67, 4.45). A history of any antenatal care visit was associated with a greater chance of a mother providing prelacteal feeds, especially visits by healthcare workers other than the local FCHV compared to the mothers who did not go for antenatal care visit (APR 1.43; 95% CI 1.11, 1.84).

**Table 4:** Determinants of pre-lacteal feeding among infants in Nepal, 2013^a,b,c^

Determinants	*n*	Fed prelacteal feeds (%)	Model 1 (Unadjusted PR) PR (95% CI)	Model 3^d^ (Adjusted PR) APR (95% CI)
Overall	1006	329 (32.7)		
Child factors				
Child’s gender				
Male	537	177(33.0)	1.00	–
Female	469	152(32.4)	1.03 (0.89–1.19)	
Child’s birth order				
First born	534	205 (38.4)	1.00	1.00
Second or later born	472	124 (26.3)	0.65 (0.53–0.81)**	0.72 (0.60,0.86)**
Breastfed within one hour of birth				
No	582	251 (43.1)	1.00	1.00
Yes	421	78 (18.5)	0.46(0.34,0.62)**	0.5 (0.37,0.67)**
Child fed colostrum				
No	164	74 (45.1)	1.00	1.00
Yes	839	255 (30.4)	0.78 (0.63,0.96)*	0.78 (0.65,0.93)*
Predominant breastfeeding (Infant < 6 mo)				
No	195	104 (53.3)	1.00	1.00
Yes	257	43 (16.7)	0.49 (0.34,0.71)**	0.51 (0.36,0.72)**
Maternal factors				
Mother’s education				
None	477	156 (32.7)	1.00	1.00
Some primary	135	46 (34.1)	1.06 (0.84–1.33)	0.92 (0.72–1.17)
Secondary and above	393	126 (32.1)	1.15 (0.91–1.46)	0.90 (0.70–1.15)
Visit by FCHVs for ANC				
No	906	285 (31.5)	1.00	1.00
Yes	100	44 (44.0)	1.17 (0.92–1.50)	1.14 (0.87–1.50)
Visit by more highly trained healthcare providers^e^ for ANC				
No	967	308 (31.9)	1.00	1.00
Yes	39	21 (53.9)	1.28 (0.90–1.81)	1.43 (1.11–1.84)*
Household factors				
Household wealth quintile				
1 (Poorest)	202	53 (26.2)	1.00	1.00
2	197	71 (36)	1.30 (0.94–1.80)	1.25 (0.9–1.73)
3	202	57 (28.2)	1.08 (0.79–1.48)	1.05 (0.75–1.47)
4	205	65 (31.7)	1.20 (0.88–1.65)	1.07 (0.78–1.46)
5 (Richest)	200	83 (41.5)	1.59 (1.13–2.25)*	1.45 (0.98–2.14)
Household head’s education				
None	480	146 (30.4)	1.00	1.00
Some primary	189	71 (37.6)	1.27 (1.01,1.59)*	1.19 (0.92–1.52)
Secondary and above	337	112 (33.2)	1.24 (0.99–1.55)	1.17 (0.92–1.48)
Cultivable land size (in Ha)				
Landless (< 0.1)	421	121 (28.7)	1.00	1.00
Small size (≥ 0.1 & < 0.5)	259	81 (31.3)	1.20 (0.98–1.47)	1.18 (0.97–1.43)
Large size (≥ 0.5)	326	127 (39)	1.26 (1.00–1.58)	1.21 (0.96–1.52)
Contextual factors				
Agro-ecological zones				
Mountain	160	23 (14.4)	–	1.00
Hill	257	67 (26.1)		1.49 (0.83–2.65)
Terai	589	239 (40.6)		2.72 (1.67–4.45)**

^a^For interpretation purposes, a PR > 1 indicates that prelacteal feeding was more likely and PR < 1 indicates that prelacteal feeding was less likely

^b^*P-value < 0.05, **P-value < 0.001

^c^(Model 2 shown in Additional file 3)

^d^Model 3 included mother’s education and visit by FCHVs for ANC as a priori covariates plus all variables that were significant (*p* < 0.2) in the first set of multivariable models

^e^“more highly trained healthcare providers” includes government health workers (MCHW/VHW, HA/AHW, Nurse/Midwife), doctors/pharmacists and NGO health workers

### Determinants of predominant breastfeeding under six months (Table 5)

Compared to infants < 2 months of age, infants of age 2 to 3.9 months and 4 to 5.9 months were 24% (APR 0.86; 95% CI 0.75, 0.98) and 43% (APR 0.57; 95% CI 0.42, 0.77) less likely to be predominantly breastfed, respectively. Children of mothers who visited health facilities for antenatal care visits were 19% (APR 1.19; 95% CI 1.02, 1.38) more likely to predominantly breastfeed than those who did not visit health facilities for antenatal care visit. Compared to women without knowledge, those women who had knowledge of exclusive breastfeeding for infants up to 6 months of age were 19% more likely to report predominantly breastfeeding their infants (APR 1.19; 95% CI 1.01, 1.39). However, paradoxically, those with knowledge of the need to breastfeed through diarrheal episodes were 20% less likely to predominantly breastfeed than those without the knowledge (APR 0.80; 95% CI 0.66, 0.97). Children from lower caste families were 47% more likely to predominantly breastfeed compared to the upper caste families (APR 1.47; 95% CI 1.02, 2.12). Those infants in the second lowest fifth of the constructed wealth index had a 32% lower chance of predominant breastfeeding compared with infants born into the poorest 20% of households (APR 0.68; 95% CI 0.51, 0.91). Compared to the children living in the mountains, infants born in households in the Hills were 33% less likely to be predominantly breastfed (APR 0.67; 95% CI 0.49, 0.93) (Table 5).

**Table 5:** Determinants of predominant breastfeeding among children less than 6 months of age in Nepal, 2013^a,b,c^

Determinants	*n*	Predominantly breastfed, *n* (%)	Model 1 (Unadjusted PR) PR (95% CI)	Model 3^d^ (Adjusted PR) APR (95% CI)
Overall	458	262 (57.2)		
Child factors				
Child’s gender				
Male	247	141 (57.1)	1.00	–
Female	211	121 (57.4)	1.01 (0.86–1.18)	
Age (in months)				
0 to 1.9	127	94 (74.0)	1.00	1.00
2 to 3.9	171	108 (63.2)	0.84 (0.73–0.98)*	0.86 (0.75–0.98)*
4 to 5.9	160	60 (37.5)	0.50 (0.37–0.68)**	0.57 (0.42–0.77)**
Child’s birth order				
First born child	228	118 (51.8)	1.00	–
Second or later born child	230	114 (62.6)	1.18 (1.00–1.40)	
Breastfed within one hour of birth				
No	251	130 (51.8)	1.00	–
Yes	205	130 (63.4)	1.23 (1.01,1.5)*	
Child fed colostrum				
No	73	43 (58.9)	1.00	–
Yes	383	217 (56.7)	1 (0.83,1.19)	
Child fed prelacteal feeds				
No	305	214 (70.2)	1.00	1.00
Yes	147	43 (29.3)	0.41 (0.29,0.57)**	0.45 (0.32,0.62)**
Maternal factors				
Mother’s education				
None	206	124 (60.2)	1.00	1.00
Some primary	66	40 (60.6)	1.03 (0.83–1.28)	1.01 (0.82–1.26)
Secondary and above	186	98 (52.7)	0.91 (0.73–1.13)	0.92 (0.76–1.13)
Visit by FCHV for ANC				
No	397	222 (55.9)	1.00	1.00
Yes	61	40 (65.6)	1.14 (0.91–1.42)	1.11 (0.90–1.38)
Visit to health facilities for ANC				
No	127	65 (51.2)	1.00	1.00
Yes	331	197 (59.5)	1.18 (1.00–1.40)	1.19 (1.02–1.38)*
Visit by FCHV for postnatal care				
No	400	224 (56)	1.00	–
Yes	58	38 (65.5)	1.26 (0.98–1.61)	
Visit to health facilities for postnatal care				
No	275	154 (56.0)	1.00	1.00
Yes	183	108 (59.0)	1.12 (0.96–1.32)	1.00 (0.87–1.15)
Maternal knowledge present on				
Exclusive breastfeeding for infants up to 6 months of age				
No	152	80 (52.6)	1.00	1.00
Yes	306	182 (59.5)	1.19 (1.03–1.39)*	1.19 (1.01–1.39)*
Breastfeeding for children during diarrhea				
No	370	222 (60.0)	1.00	1.00
Yes	88	40 (45.5)	0.72 (0.59–0.88)*	0.80 (0.66–0.97)*
Women’s empowerment (scale: 0–14, Md = 5)				
< = 8 (less empowered)	377	212 (56.2)	1.00	–
> = 9 (more empowered)	81	50 (61.7)	1.15 (0.93–1.42)	
Household factors				
Ethnicity/Caste				
Upper caste	111	55 (49.6)	1.00	1.00
Disadvantaged non-dalit Terai caste	156	100 (64.1)	1.26 (0.85–1.86)	1.38 (0.89–2.14)
Janajatis	101	44 (43.6)	0.95 (0.66–1.36)	1.02 (0.75–1.39)
Lower caste^e^	90	63 (70.0)	1.48 (1.09–2.00)*	1.47 (1.02–2.12)*
Household wealth quintile				
1 (Poorest)	79	53 (67.1)	1.00	1.00
2	91	47 (51.7)	0.66 (0.49–0.91)*	0.68 (0.51–0.91)*
3	86	50 (58.1)	0.77 (0.61–0.99)*	0.79 (0.62–1.00)
4	100	61 (61.0)	0.82 (0.64–1.05)	0.87 (0.7–1.07)
5 (Richest)	102	51 (50.0)	0.71 (0.49–1.04)	0.79 (0.58–1.08)
Household head’s education				
None	205	122 (59.5)	1.00	1.00
Some primary	102	51 (50)	0.85 (0.69–1.05)	0.98 (0.8–1.19)
Secondary and above	151	89 (58.9)	0.99 (0.82–1.19)	1.14 (0.94–1.37)
Community level factors				
Agro-ecological zones				
Mountain	83	52 (62.7)	–	1.00
Hill	116	47 (40.5)		0.67 (0.49–0.93)*
Terai	259	163 (62.9)		1.06 (0.76–1.48)

^a^For interpretation purposes, a PR > 1 indicates children were more likely to be predominantly breastfed and PR < 1 indicates children were less likely

^b^*P-value < 0.05, **P-value < 0.001

^c^(Model 2 shown in Additional file 4)

^d^Model 3 included mother’s education and visit by FCHVs for ANC as a priori covariates plus all variables that were significant (*p* < 0.2) in the first set of multivariable models

^e^“Lower caste” includes Dalits and religious minorities

### Coexistence of breastfeeding practice indicators

This study also assessed inter-relationships between breastfeeding practices. Compared to infants not fed prelacteal feeds, infants given prelacteal feeds were 51% less likely to be breastfed within the first hour of birth (APR 0.49; 95% CI 0.36, 0.66) and 55% less likely to be predominantly breastfed through 6 months of age (APR 0.45; 95% CI 0.32, 0.62). Infants reported to have received colostrum were 26% more likely to have started breastfeeding within an hour of birth (APR 1.26; 95% CI 1.04, 1.54) compared to those who did not receive colostrum. Compared to infants who were not breastfed within an hour of birth, infants breastfed within 1 h of birth have 50% less chance of being fed prelacteal feeds (APR 0.50; 95% CI 0.37, 0.67).

## Discussion

This study profiles the prevalence, quality and determinants of breastfeeding practices in a national sample of infants in Nepal. While breastfeeding is nearly universal, most mothers delay introduction of breastmilk by an hour or more after delivery. Our estimates of the percentage of mothers introducing breastmilk within 1 h of birth was lower (41.8% vs. 54.9%), and prelacteal feeding slightly higher (32.7 vs. 28.6%) than reported in the 2016 Demographic and Health Survey (DHS) . This difference might be due, in part, to different recall periods, with the DHS including children born in the 2 years preceding the survey without regard to vital status at the time of interview. Local area sampling variability and variation in the way questions were coded may also lead to differences in estimates. As the DHS does not report colostrum feeding, our estimate that 83.5% of mothers fed colostrum at some time in the early breastfeeding experience provides a first national estimate of this practice. Due to differences in definitions, our estimates of predominant breastfeeding are not directly comparable to DHS estimates of exclusive breastfeeding, as we did not collect information on intake of fluids and, under an assumption that the transition from exclusive breastfeeding to inclusion of other food items may initially be sporadic and a single 24 h recall period overestimates the prevalence [42], we used a recall period of 7 days while DHS used a 24 h recall period. While the lack of information on exclusive breastfeeding prevalence in our population is a study limitation, predominant breastfeeding can still serve as an important breastfeeding indicator when information on exclusive breastfeeding is not available [32]. For an instance, a study done in Mexico showed that predominant breastfeeding is associated with lower gastrointestinal infection among infants at 6 months of age [43]. Another prospective cohort study done in Brazil showed that predominant breastfeeding increased the growth rate of infants in the first months of life [44].

Our findings suggest that introducing prelacteal feeds may disrupt the feeding of colostrum and increase the likelihood of other foods being introduced in the first 6 months, as has been reported in Ethiopia and other settings [45]. Prelacteal feeding has also been associated with delayed initiation of breastfeeding in Bangladesh [19]. In Nepal, prelacteal feeding has been associated with a higher risk of infant mortality in a dose-response manner [2], adding a compelling evidence for the need to breastfeed and avoid prelacteal feeds immediately after birth.

Our results reveal geographical differences in breastfeeding practices within Nepal. Feeding colostrum was less prevalent while the introduction of prelacteal feeds was more prevalent in the Terai. These observations are consistent with an earlier analysis suggesting that timely initiation of breastfeeding was 42% less likely in the Terai than mountains [46]. Ethnicity/caste was also associated with breastfeeding practices, with children from lower caste families being more likely to be predominantly breastfeed than upper caste families, possibly because the latter were more wealthy and able to afford breast milk substitutes. In the present study, mothers under 20 years of age were more likely to report timely initiation of breastfeeding, defined as breastfeeding within 1 h after birth, than older mothers, but less likely to report feeding their infants colostrum. Further research may might reveal reasons underlying differences in practice, including varied traditions among ethnic groups.

Infant age at assessment was an important predictor of predominant breastfeeding, with older children more likely to have already had semi-solid or solid foods introduced. This finding is consistent with findings from other studies in South Asia [47–49] and elsewhere [50–52] indicating a transition to complementary feeding in mid-infancy.

Maternal education did not appear to exert a strong influence on breastfeeding practices, unlike in Nepal [53], Bangladesh [54], India [55] and Pakistan [56] where women without formal education have been more likely to report a delay in the initiation of breastfeeding. An explanation may be irrespective of maternal education level and socioeconomic status if the mothers undergo caesarian section, they are less likely to initiate early breastfeeding [57]. Paradoxically, mothers having knowledge of the need to breastfeed through diarrheal episodes were less likely to predominantly breastfeed, possibly reflecting common occurrence of infantile diarrhea and an understood need to feed other fluids or foods during diarrhea.

Mothers engaged in agricultural occupations were more likely introduce breastfeeding shortly after birth, as seen elsewhere in Nepal [53]. In contrast, in households headed by salaried or agriculture workers, infants were less likely to receive colostrum. Reasons for different infant feeding patterns by occupation remain largely unknown and merit further exploration.

Households below the 20th percentile of our derived wealth index reported a higher prevalence of predominant breastfeeding than all wealthier groups, a finding that is consistent with studies from India [48] and Sri Lanka [58]. Possibly, wealthier women may be salaried workers, such as teachers, working in shops or self-employed (data not shown), thus finding it more difficulty to exclusively/predominantly breastfeed. On the other hand, households above the 80th percentile of the wealth index were more likely to feed colostrum to their newborns, consistent with practices observed in the District Level Household Survey (DLHS-3) of India where infants from richer households in non-Empowered Action Group States were more likely than less wealthy homes to feed colostrum [59].

First born children were more likely to be fed prelacteal feeds than later siblings, consistent with observations from the 2011 NDHS [33]. In contrast, in Rupandehi District of Nepal, the odds of giving prelacteal feeds increased with parity [28], revealing possible variation in prelacteal feeding across Nepal, as has been observed with child feeding [60]. Surprisingly, mothers who reported receiving antenatal care from formally trained government health workers, doctors, pharmacists and NGO health workers were also more likely to give their infants prelacteal feeds, a pattern not observed with home visits from FCHVs. Visits to more highly trained providers may be have been due to maternal illness or obstetric emergencies (e.g., requiring caesarian section) making it difficult for mothers to initiate breastfeeding [28, 61]. However, it has also been shown in Nepal that recommendations to mothers to use a breastmilk substitute from a health worker increases the likelihood of compliance with this practice than if no such guidance is given [62]. On the other hand, counseling during ANC about the importance of breastfeeding can influence a mother to initiate early breastfeeding [63]. Our findings provide support for continuing this approach in Nepal. Finally, the practice of feeding prelacteal feeds was more common in the Terai, consistent with observations from the NDHS [33], clearly identifying this region as one of high priority for intensified efforts to change this practice.

The main strengths of this study are that the sampling frame was designed to both statistically represent the country as well as the three major agro-ecological zones and that the survey content included a wide variety of potential determinants. Limitations of the study include its cross-sectional design and the reliance on predominant breastfeeding rather than exclusive breastfeeding as an outcome indicator, due to the lack of inclusion of plain water and other liquids in the 7-day recall. Additionally, we cannot rule out the possibility of social desirability bias or potential survival bias given the reliance on recall-based indicators and strong associations between breastfeeding and the risk of infant mortality.

## Conclusions

Our study affirms a need to continue improving breastfeeding practices in rural Nepal through strengthened antenatal care and IYCF practices. Of particular concern is the need to reduce prelacteal feeding, especially in the southern plains (Terai) and encourage early introduction of breastfeeding, both of which may help extend the duration of predominant breastfeeding, and likely, exclusive breastfeeding. Increasing coverage of ANC check-ups and focusing efforts on early IYCF practices may be a useful way of improving coverage.

## Additional files

Additional file 1: Determinants of breastfeeding within one hour of birth among infants in Nepal, 2013. This file contains model 2 in addition to other models presented in the main text. (PDF 153 kb)

Additional file 2: Determinants of feeding colostrum among infants in Nepal, 2013. This file contains model 2 in addition to other models presented in the main text. (PDF 183 kb)

Additional file 3: Determinants of prelacteal feeding among infants in Nepal, 2013. This file contains model 2 in addition to other models presented in the main text. (PDF 140 kb)

Additional file 4: Determinants of predominant breastfeeding among children less than 6 months of age in Nepal, 2013. This file contains model 2 in addition to other models presented in the main text (PDF 154 kb)

## Abbreviations

AHW: Auxiliary Health Worker
ANC: Antenatal care
APR: Adjusted Prevalence Ratio
CI: Confidence Interval
DHS: Demographic and Health Survey
FCHV: Female Community Health Volunteer
HA: Health Assistant
IYCF: Infant and Young Child Feeding
MCHW: Maternal and Child Health Worker
ORS: Oral Rehydration Solution
PHC: Primary Health Center
VDC: Village Development Committee
VHW: Village Health Worker
WHO: World Health Organization
