# Supplementary material for: Determinants of infant breastfeeding practices in Nepal: a national study
Source: Int Breastfeed J. 2019 Apr 3;14:14. doi: 10.1186/s13006-019-0208-y (PMC6448244; doi:10.1186/s13006-019-0208-y)
Supplement: Supplementary file 1 — Determinants of breastfeeding within one hour of birth among infants in Nepal, 2013. This file contains model 2 in addition to other models presented in the main text. (PDF 153 kb) [file 13006_2019_208_MOESM1_ESM.pdf]

Additional file 1 Determinants of breastfeeding within one hour of birth among infants in Nepal, 2013[a,b]

**Determinants**	n (%)	Breastfed within an hour, n(%)	Model 1 (Unadjusted PR) PR (95% CI)	Model 2[c] (Adjusted PR) APR (95% CI)	Model 3[d] (Adjusted PR) APR (95% CI)
**Overall**	1011 (100)	423 (41.8)			
**Child factors**					
Child's sex					
Male	541 (53.5)	222(41.0)	1.00	-	-
Female	470 (46.5)	201(42.8)	1.03 (0.91-1.18)		
Child's birth order					
First born	538 (53.2)	210 (39.0)	1.00	1.00	-
Second or later born	473 (46.8)	213 (45.0)	1.18 (1.00,1.38)*	1.05 (0.91,1.23)	
Child fed colostrum					
No	167 (16.5)	54 (32.3)	1.00	1.00	1.00
Yes	844 (83.5)	369 (43.7)	1.32 (1.07,1.64)*	1.24 (1.02,1.51)*	1.26 (1.04,1.54)*
Child fed prelacteals					
No	677 (67.3)	343 (50.9)	1.00	1.00	1.00
Yes	329 (32.7)	78 (23.7)	0.47 (0.35,0.63)**	0.49 (0.36,0.67)**	0.49 (0.36,0.66)**
Predominant breastfeeding (Infant <6 mo)					
No	196 (42.8)	75 (38.3)	1.00	1.00	-
Yes	262 (57.2)	130 (50.0)	1.33 (1.01,1.74)*	1.13 (0.95,1.34)	
**Maternal factors**					
Mother's education					
None	479 (47.4)	200 (41.8)	1.00	1.00	1.00
Some primary	135 (13.4)	53 (39.3)	0.93 (0.72,1.2)	0.87 (0.68,1.12)	0.91 (0.70-1.19)
Secondary and above	396 (39.2)	170 (42.9)	1.00 (0.87,1.16)	0.96 (0.83,1.12)	1.01 (0.84-1.21)
Mother's age (in years)					
15-19.9	149 (14.7)	68 (45.6)	1.00	1.00	1.00
20-29.9	697 (68.9)	299 (42.9)	0.93 (0.79,1.09)	0.79 (0.67,0.93)*	0.81 (0.68-0.95)*
≥30	165 (16.3)	56 (33.9)	0.72 (0.53,0.97)*	0.57 (0.40,0.81)*	0.61 (0.43-0.87)*
Mother's occupation					
Unemployed	773 (76.5)	305 (39.5)	1.00	1.00	1.00
Agriculture	136 (13.5)	72 (52.9)	1.31 (1.04,1.64)*	1.27 (1.04,1.56)*	1.28 (1.02-1.60)*
Other employment[e]	102 (10.1)	46 (45.1)	1.10 (0.85,1.43)	1.11 (0.86,1.44)	1.09 (0.83-1.42)
Visit by FCHVs for ANC					
No	909 (89.9)	382 (42)	1.00	1.00	
Yes	102 (10.1)	41 (40.2)	0.98 (0.78,1.24)	0.94 (0.74-1.20)	0.99 (0.77-1.27)
Visit by FCHVs for post-natal care					
No	903 (89.3)	371 (41.1)	1.00	1.00	1.00
Yes	108 (10.7)	52 (48.2)	1.17 (0.94,1.45)	1.17 (0.95,1.43)	1.12 (0.91-1.37)
Visit by more highly trained health care providers[e] for post-natal care					

No	959 (94.9)	408 (42.5)	1.00	1.00	1.00
Yes	52 (5.1)	15 (28.9)	0.69 (0.49,0.99)*	0.69 (0.49,0.98)*	0.72 (0.49-1.05)
Maternal knowledge on exclusive breastfeeding for infants up to 6 months of age					
No	368 (36.4)	136 (37)	1.00	1.00	1.00
Yes	643 (63.6)	287 (44.6)	1.17 (0.97,1.41)	1.18 (0.98,1.43)	1.19 (0.99-1.44)
Number of live births given					
1	380 (37.7)	149 (39.2)	1.00	1.00	1.00
>=2	629 (62.3)	274 (43.6)	1.12 (0.96,1.30)	1.24 (1.02,1.49)*	1.11 (0.85-1.43)
**Household factors**					
Ethnicity/Caste					
Upper caste	216 (21.4)	104 (48.2)	1.00	1.00	1.00
Disadvantaged non-dalit Terai caste	341 (33.7)	137 (40.2)	0.95 (0.76,1.20)	1.01 (0.80,1.27)	1.02 (0.81-1.27)
Janajatis	227 (22.5)	87 (38.3)	0.83 (0.67,1.04)	0.86 (0.69,1.08)	0.91 (0.73-1.14)
Lower caste[e]	227 (22.5)	95 (41.9)	0.93 (0.74,1.17)	0.95 (0.75,1.2)	0.95 (0.76-1.19)
Household wealth quintile					
1 (Poorest)	202 (20)	89 (44.1)	1.00	1.00	1.00
2	198 (19.6)	79 (39.9)	0.92 (0.73,1.14)	0.93 (0.74,1.16)	0.94 (0.75-1.18)
3	204 (20.2)	93 (45.6)	1.03 (0.8,1.33)	1.07 (0.83,1.39)	1.07 (0.82-1.38)
4	204 (20.2)	93 (45.6)	1.05 (0.82,1.34)	1.05 (0.81,1.36)	1.07 (0.79-1.45)
5 (Richest)	203 (20.1)	69 (34.0)	0.77 (0.57,1.06)	0.75 (0.53,1.06)	0.79 (0.55-1.12)
Occupation of household head					
Unemployed[e]	122 (12.1)	51 (41.8)	1.00	1.00	1.00
Wage employment	190 (18.8)	83 (43.7)	1.07 (0.83,1.39)	1.06 (0.81,1.39)	1.03 (0.81-1.33)
Business/self-employment	210 (20.8)	72 (34.3)	0.82 (0.58,1.17)	0.83 (0.57,1.2)	0.80 (0.57-1.13)
Salaried worker	101 (10)	57 (56.4)	1.32 (1.01,1.73)*	1.31 (0.96,1.77)	1.27 (0.94-1.71)
Agriculture	387 (38.3)	160 (41.3)	0.99 (0.73,1.34)	0.93 (0.68,1.27)	0.88 (0.65-1.19)
**Contextual factors**					
Agro-ecological zones					
Mountain	158 (15.6)	77(48.7)	-	-	1.00
Hill	256 (25.3)	111(43.4)			1.00 (0.79-1.26)
Terai	597 (59.1)	235(39.4)			1.03 (0.78-1.36)

[a] For interpretation purposes, a PR >1 indicates children are more likely to be breastfed within an hour of birth and PR<1 indicates children are less likely.
[b] * P-value <0.05, ** P-value <0.001.
[c] Model 2 included mother's education and visit by FCHVs for ANC as a priori covariates and maternal variables from unadjusted analysis with a p<0.2.
[d] Model 3 included mother's education and visit by FCHVs for ANC as a priori covariates plus all variables that were significant (p<0.2) in the first set of multivariable models.
[e] "Other employment" included wage employment, salaried worker and Business/self-employment. "More highly trained health care providers" includes government health workers (MCHW/VHW, HA/AHW, Nurse/Midwife), doctors/pharmacists and NGO health workers. "Lower caste" includes Dalits and religious minorities. "Unemployed" includes student, non-earning occupation as well as non-working.
