# Supplementary material for: Determinants of infant breastfeeding practices in Nepal: a national study
Source: Int Breastfeed J. 2019 Apr 3;14:14. doi: 10.1186/s13006-019-0208-y (PMC6448244; doi:10.1186/s13006-019-0208-y)
Supplement: Supplementary file 2 — Determinants of feeding colostrum among infants in Nepal, 2013. This file contains model 2 in addition to other models presented in the main text. (PDF 183 kb) [file 13006_2019_208_MOESM2_ESM.pdf]

Additional file 2 Determinants of feeding colostrum among infants in Nepal, 2013[a,b]

Determinants	n	Fed colostrum, n (%)	Model 1 (Unadjusted PR) PR (95% CI)	Model 2[c] (Adjusted PR) APR (95% CI)	Model 3[d] (Adjusted PR) APR (95% CI)
Overall	1011	844 (83.5)			
**Child factors**					
Child's sex					
Male	541	448(82.8)	1.00	-	-
Female	470	396(84.3)	1.01 (0.96-1.06)		
Child's birth order					
First born	538	455 (84.6)	1.00	-	-
Second or later born	473	389 (82.2)	0.99 (0.93-1.06)		
Breastfed within one hour of birth					
No	588	475 (80.8)	1.00	1.00	1.00
Yes	423	369 (87.2)	1.07 (1.01,1.13)*	1.05 (1.01,1.1)*	1.06(1.01,1.11)*
Child fed prelacteals					
No	674	584 (86.7)	1.00	1.00	1.00
Yes	329	255 (77.5)	0.92 (0.84,1.00)*	0.93(0.86,1.01)	0.92(0.86,0.99)*
Predominant breastfeeding (Infant <6 mo)					
No	196	166 (84.7)	1.00	-	-
Yes	260	217 (83.5)	1.00 (0.94,1.05)		
**Maternal factors**					
Mother's education					
None	479	373 (77.9)	1.00	1.00	1.00
Some primary	135	115 (85.2)	1.08 (1-1.17)	1.07 (0.99-1.17)	1.05 (0.96-1.14)
Secondary and above	396	355 (89.7)	1.12 (1.06-1.19)**	1.09 (1.03-1.16)*	1.04 (0.97-1.12)
Mother's age (in years)					
15-19.9	149	113 (75.8)	1.00	1.00	1.00
20-29.9	697	595 (85.4)	1.12 (1.02-1.23)*	1.11 (1.02-1.21)*	1.09 (1.00-1.19)*
≥30	165	136 (82.4)	1.05 (0.93-1.19)	1.1 (0.96-1.25)	1.07 (0.94-1.22)
Visit by FCHVs for ANC					
No	909	760 (83.6)	1.00	1.00	1.00
Yes	102	84 (82.4)	1.01 (0.91-1.13)	1.01 (0.92-1.12)	1 (0.91-1.11)
Visit by more highly trained health care providers[e] for ANC					
No	972	816 (84.0)	1.00	1.00	1.00
Yes	39	28 (71.8)	0.91 (0.8-1.02)	0.89 (0.81,0.98)*	0.94 (0.86-1.03)
Visit to health facilities for ANC					
No	388	308 (79.4)	1.00	1.00	1.00
Yes	623	536 (86.0)	1.08 (1.00-1.17)	1.07 (0.98-1.15)	1.07 (0.98-1.16)

Maternal knowledge on exclusive breastfeeding for infants up to 6 months of age					
No	368	285 (77.5)	1.00	1.00	1.00
Yes	643	559 (86.9)	1.08 (1.02-1.15)*	1.04 (0.98-1.10)	1.04 (0.98-1.09)
Women's empowerment (scale: 0-14, Md=5)					
<u><</u>8 (less empowered)	826	677 (82.0)	1.00	1.00	1.00
<u>></u>9 (more empowered)	185	167 (90.3)	1.07 (1.00-1.14)*	1.05 (0.99-1.12)	1.08 (1.01-1.15)*
Had abortions in lifetime					
No	972	806 (82.9)	1.00	1.00	1.00
Yes	39	38 (97.4)	1.12 (1.06-1.19)**	1.10 (1.03-1.18)*	1.10 (1.02-1.17)*
Had miscarriage/still births in lifetime					
No	847	713 (84.2)	1.00	1.00	1.00
Yes	164	131 (79.9)	0.94 (0.88-1.00)	0.93 (0.87-1.00)	0.93 (0.87-1.00)
**Household factors**					
Household head's education					
None	484	381 (78.7)	1.00	1.00	1.00
Some primary	189	159 (84.1)	1.06 (0.98-1.14)	1.01 (0.93,1.1)	1.01 (0.94-1.09)
Secondary and above	338	304 (89.9)	1.12 (1.07-1.17)**	1.07 (1.02,1.12)*	1.05 (1.00-1.10)
Household wealth quintile					
1 (Poorest)	202	158 (78.2)	1.00	1.00	1.00
2	198	156 (78.8)	1.02 (0.92-1.13)	1.01 (0.9,1.12)	1 (0.90-1.1)
3	204	162 (79.4)	1.02 (0.94-1.10)	1 (0.92,1.08)	0.97 (0.90-1.06)
4	204	183 (89.7)	1.17 (1.08-1.26)**	1.13 (1.04,1.22)*	1.09 (1.01-1.19)*
5 (Richest)	203	185 (91.1)	1.17 (1.10-1.25)**	1.13 (1.05,1.22)**	1.08 (1.00-1.18)
Occupation of household head					
Unemployed[e]	122	107 (87.7)	1.00	1.00	1.00
Wage employment	190	152 (80)	0.94 (0.86-1.03)	0.96 (0.89,1.05)	0.96 (0.89-1.05)
Business/self-employment	210	176 (83.8)	0.97 (0.90-1.05)	0.96 (0.89,1.03)	0.94 (0.88-1.00)
Salaried worker	101	88 (87.1)	0.98 (0.91-1.06)	0.93 (0.86,1.01)	0.91 (0.84-0.98)*
Agriculture	387	320 (82.7)	0.94 (0.88-1.02)	0.92 (0.85,1.00)*	0.91 (0.84-0.98)*
Cultivable land size (in Ha)					
Landless (<0.1)	421	343 (81.5)	1.00	1.00	1.00
Small size (<u>></u>0.1 & <0.5)	260	216 (83.1)	1 (0.93-1.08)	1.05 (0.97,1.14)	1.06 (0.99-1.15)
Large size (<u>></u>0.5)	330	285 (86.4)	1.07 (1.01-1.14)*	1.1 (1.04,1.17)**	1.12 (1.05-1.19)**
**Contextual factors**					
Agro-ecological zones					
Mountain	158	144 (91.1)	-	-	1.00
Hill	256	231 (90.2)			0.99 (0.93-1.05)
Terai	597	469 (78.6)			0.9 (0.83-0.97)*
Ward infrastructure is more developed					

No	502	394 (78.5)	1.00	1.00	1.00
Yes	509	450 (88.4)	1.09 (1.00-1.18)*	1.09 (1.00-1.18)*	1.04 (0.96-1.13)

[a] Prevalence ratio: a PR >1 indicates feeding of colostrum is more likely and PR <1 indicates that feeding of colostrum is less likely.

[b] * P-value <0.05, ** P-value <0.001.

[c] Model 2 included mother's education and visit by FCHVs for ANC as *a priori* covariates and maternal variables from unadjusted analysis with a p<0.2.

[d]Model 3 included mother's education and visit by FCHVs for ANC as *a priori* covariates plus all variables that were significant (p<0.2) in the first set of multivariable models.

[e] "more highly trained health care providers" includes government health workers (MCHW/VHW, HA/AHW, Nurse/Midwife), doctors/pharmacists and NGO health workers. "Unemployed" includes student, non-earning occupation as well as non-working.
