# Supplementary material for: Determinants of infant breastfeeding practices in Nepal: a national study
Source: Int Breastfeed J. 2019 Apr 3;14:14. doi: 10.1186/s13006-019-0208-y (PMC6448244; doi:10.1186/s13006-019-0208-y)
Supplement: Supplementary file 3 — Determinants of prelacteal feeding among infants in Nepal, 2013. This file contains model 2 in addition to other models presented in the main text. (PDF 140 kb) [file 13006_2019_208_MOESM3_ESM.pdf]

Additional file 3 Determinants of prelacteal feeding among infants in Nepal, 2013[a,b]

**Determinants**	n	Fed prelacteals, n (%)	Model 1 (Unadjusted PR) PR (95% CI)	Model 2[c] (Adjusted PR) APR (95% CI)	Model 3[d] (Adjusted PR) APR (95% CI)
Overall	1006	329 (32.7)			
**Child factors**					
Child's sex					
Male	537	177(33.0)	1.00	-	-
Female	469	152(32.4)	1.03 (0.89-1.19)		
Child's birth order					
First born	534	205 (38.4)	1.00	1.00	1.00
Second or later born	472	124 (26.3)	0.65 (0.53-0.81)**	0.70 (0.58,0.84)**	0.72 (0.60,0.86)**
Breastfed within one hour of birth					
No	582	251 (43.1)	1.00	1.00	1.00
Yes	421	78 (18.5)	0.46 (0.34,0.62)**	0.5 (0.37,0.67)**	0.50 (0.37,0.67)**
Child fed colostrum					
No	164	74 (45.1)	1.00	1.00	1.00
Yes	839	255 (30.4)	0.78 (0.63,0.96)*	0.83 (0.67,1.02)	0.78 (0.65,0.93)*
Predominant breastfeeding (Infant <6 mo)					
No	195	104 (53.3)	1.00	1.00	1.00
Yes	257	43 (16.7)	0.49 (0.34,0.71)**	0.53 (0.38,0.76)**	0.51 (0.36,0.72)**
**Maternal factors**					
Mother's education					
None	477	156 (32.7)	1.00	1.00	1.00
Some primary	135	46 (34.1)	1.06 (0.84-1.33)	1.00 (0.79-1.26)	0.92 (0.72-1.17)
Secondary and above	393	126 (32.1)	1.15 (0.91-1.46)	1.06 (0.84-1.34)	0.90 (0.70-1.15)
Visit by FCHVs for ANC					
No	906	285 (31.5)	1.00	1.00	1.00
Yes	100	44 (44.0)	1.17 (0.92-1.50)	1.19 (0.92-1.54)	1.14 (0.87-1.50)
Visit by more highly trained health care providers[e] for ANC					
No	967	308 (31.9)	1.00	1.00	1.00
Yes	39	21 (53.9)	1.28 (0.90-1.81)	1.32 (0.93-1.86)	1.43 (1.11-1.84)*
**Household factors**					
Household wealth quintile					
1 (Poorest)	202	53 (26.2)	1.00	1.00	1.00
2	197	71 (36)	1.30 (0.94-1.80)	1.26 (0.91-1.75)	1.25 (0.9-1.73)
3	202	57 (28.2)	1.08 (0.79-1.48)	1.05 (0.76-1.44)	1.05 (0.75-1.47)
4	205	65 (31.7)	1.20 (0.88-1.65)	1.13 (0.83-1.53)	1.07 (0.78-1.46)
5 (Richest)	200	83 (41.5)	1.59 (1.13-2.25)*	1.48 (1.04-2.11)*	1.45 (0.98-2.14)
Household head's education					
None	480	146 (30.4)	1.00	1.00	1.00

Some primary	189	71 (37.6)	1.27 (1.01,1.59)*	1.17 (0.93-1.47)	1.19 (0.92-1.52)
Secondary and above	337	112 (33.2)	1.24 (0.99-1.55)	1.11 (0.87-1.40)	1.17 (0.92-1.48)
Cultivable land size (in Ha)					
Landless (<0.1)	421	121 (28.7)	1.00	1.00	1.00
Small size (≥0.1 & <0.5)	259	81 (31.3)	1.20 (0.98-1.47)	1.21 (0.99-1.49)	1.18 (0.97-1.43)
Large size (≥0.5)	326	127 (39)	1.26 (1.00-1.58)	1.23 (0.98-1.55)	1.21 (0.96-1.52)
**Contextual factors**					
Agro-ecological zones					
Mountain	160	23 (14.4)	-	-	1.00
Hill	257	67 (26.1)			1.49 (0.83-2.65)
Terai	589	239 (40.6)			2.72 (1.67-4.45)**

[a] For interpretation purposes, a PR >1 indicates that prelacteal feeding was more likely and PR<1 indicates that prelacteal feeding was less likely.
[b] * P-value <0.05, ** P-value <0.001.
[c] Model 2 included mother's education and visit by FCHVs for ANC as a priori covariates and maternal variables from unadjusted analysis with a p<0.2.
[d] Model 3 included mother's education and visit by FCHVs for ANC as a priori covariates plus all variables that were significant (p<0.2) in the first set of multivariable models.
[e] "more highly trained health care providers" includes government health workers (MCHW/VHW, HA/AHW, Nurse/Midwife), doctors/pharmacists and NGO health workers.
