# Supplementary material for: Determinants of infant breastfeeding practices in Nepal: a national study
Source: Int Breastfeed J. 2019 Apr 3;14:14. doi: 10.1186/s13006-019-0208-y (PMC6448244; doi:10.1186/s13006-019-0208-y)
Supplement: Supplementary file 4 — Determinants of predominant breastfeeding among children less than 6 months of age in Nepal, 2013. This file contains model 2 in addition to other models presented in the main text (PDF 154 kb) [file 13006_2019_208_MOESM4_ESM.pdf]

Additional file 4 Determinants of predominant breastfeeding among children less than 6 months of age in Nepal, 2013[a,b]

**Determinants**	n	Predominantly breastfed, n(%)	Model 1 (Unadjusted PR) PR (95% CI)	Model 2[c] (Adjusted PR) APR (95% CI)	Model 3[d] (Adjusted PR) APR (95% CI)
Overall	458	262 (57.2)			
**Child factors**					
Child's sex					
Male	247	141 (57.1)	1.00	-	-
Female	211	121 (57.4)	1.01 (0.86-1.18)		
Age (in months)					
0 to 1.9	127	94 (74.0)	1.00	1.00	1.00
2 to 3.9	171	108 (63.2)	0.84 (0.73-0.98)*	0.84 (0.73-0.96)*	0.86 (0.75-0.98)*
4 to 5.9	160	60 (37.5)	0.50 (0.37-0.68)**	0.53 (0.38-0.72)**	0.57 (0.42-0.77)**
Child's birth order					
First born child	228	118 (51.8)	1.00	1.00	-
Second or later born child	230	114 (62.6)	1.18 (1.00-1.40)	1.01 (0.87-1.16)	
Breastfed within one hour of birth					
No	251	130 (51.8)	1.00	1.00	-
Yes	205	130 (63.4)	1.23 (1.01,1.5)*	1.02 (0.85,1.22)	
Child fed colostrum					
No	73	43 (58.9)	1.00	-	-
Yes	383	217 (56.7)	1 (0.83,1.19)		
Child fed prelacteals					
No	305	214 (70.2)	1.00	1.00	1.00
Yes	147	43 (29.3)	0.41 (0.29,0.57)**	0.43 (0.31,0.59)**	0.45 (0.32,0.62)**
**Maternal factors**					
Mother's education					
None	206	124 (60.2)	1.00	1.00	1.00
Some primary	66	40 (60.6)	1.03 (0.83-1.28)	1.03 (0.83-1.27)	1.01 (0.82-1.26)
Secondary and above	186	98 (52.7)	0.91 (0.73-1.13)	0.89 (0.71-1.11)	0.92 (0.76-1.13)
Visit by FCHV for ANC					
No	397	222 (55.9)	1.00	1.00	1.00
Yes	61	40 (65.6)	1.14 (0.91-1.42)	1.06 (0.84-1.35)	1.11 (0.90-1.38)
Visit to health facilities for ANC					
No	127	65 (51.2)	1.00	1.00	1.00
Yes	331	197 (59.5)	1.18 (1.00-1.40)	1.16 (0.98-1.38)	1.19 (1.02-1.38)*
Visit by FCHV for post-natal care					
No	400	224 (56)	1.00	1.00	-
Yes	58	38 (65.5)	1.26 (0.98-1.61)	1.14 (0.86-1.51)	

Visit to health facilities for post-natal care					
No	275	154 (56.0)	1.00	1.00	1.00
Yes	183	108 (59.0)	1.12 (0.96-1.32)	1.10 (0.95-1.27)	1.00 (0.87-1.15)
Maternal knowledge present on					
Exclusive breastfeeding for infants up to 6 months of age					
No	152	80 (52.6)	1.00	1.00	1.00
Yes	306	182 (59.5)	1.19 (1.03-1.39)*	1.23 (1.03-1.46)*	1.19 (1.01-1.39)*
Breastfeeding for children during diarrhea					
No	370	222 (60.0)	1.00	1.00	1.00
Yes	88	40 (45.5)	0.72 (0.59-0.88)*	0.74 (0.61-0.91)*	0.80 (0.66-0.97)*
Women's empowerment (scale: 0-14, Md=5)					
<=8 (less empowered)	377	212 (56.2)	1.00	1.00	-
>= 9 (more empowered)	81	50 (61.7)	1.15 (0.93-1.42)	1.03 (0.84-1.25)	
**Household factors**					
Ethnicity/Caste					
Upper caste	111	55 (49.6)	1.00	1.00	1.00
Disadvantaged non-dalit Terai caste	156	100 (64.1)	1.26 (0.85-1.86)	1.26 (0.87-1.83)	1.38 (0.89-2.14)
Janajatis	101	44 (43.6)	0.95 (0.66-1.36)	0.98 (0.71-1.36)	1.02 (0.75-1.39)
Lower caste[e]	90	63 (70.0)	1.48 (1.09-2.00)*	1.51 (1.11-2.06)*	1.47 (1.02-2.12)*
Household wealth quintile					
1 (Poorest)	79	53 (67.1)	1.00	1.00	1.00
2	91	47 (51.7)	0.66 (0.49-0.91)*	0.67 (0.48-0.93)*	0.68 (0.51-0.91)*
3	86	50 (58.1)	0.77 (0.61-0.99)*	0.79 (0.64-0.98)*	0.79 (0.62-1.00)
4	100	61 (61.0)	0.82 (0.64-1.05)	0.85 (0.67-1.09)	0.87 (0.7-1.07)
5 (Richest)	102	51 (50.0)	0.71 (0.49-1.04)	0.74 (0.5-1.08)	0.79 (0.58-1.08)
Household head's education					
None	205	122 (59.5)	1.00	1.00	1.00
Some primary	102	51 (50)	0.85 (0.69-1.05)	0.96 (0.78-1.19)	0.98 (0.8-1.19)
Secondary and above	151	89 (58.9)	0.99 (0.82-1.19)	1.13 (0.95-1.35)	1.14 (0.94-1.37)
**Community level factors**					
Agro-ecological zones					
Mountain	83	52 (62.7)	-	-	1.00
Hill	116	47 (40.5)			0.67 (0.49-0.93)*
Terai	259	163 (62.9)			1.06 (0.76-1.48)

[a] For interpretation purposes, a PR >1 indicates children were more likely to be predominantly breastfed and PR<1 indicates children were less likely.

[b] * P-value <0.05, ** P-value <0.001.

[c] Model 2 included mother's education and visit by FCHVs for ANC as a priori covariates and maternal variables from unadjusted analysis with a p<0.2.

[d]Model 3 included mother's education and visit by FCHVs for ANC as a priori covariates plus all variables that were significant ($p<0.2$) in the first set of multivariable models.
[e] "Lower caste" includes Dalits and religious minorities.
